# Associations between Recent Exposure to Ambient Fine Particulate Matter and Blood Pressure in the Multi-Ethnic Study of Atherosclerosis (MESA)

**DOI:** 10.1289/ehp.10899

**Published:** 2008-01-24

**Authors:** Amy H. Auchincloss, Ana V. Diez Roux, J. Timothy Dvonch, Patrick L. Brown, R. Graham Barr, Martha L. Daviglus, David C. Goff, Joel D. Kaufman, Marie S. O’Neill

**Affiliations:** 1 Department of Epidemiology and; 2 Department of Environmental Health Sciences, School of Public Health, University of Michigan, Ann Arbor, Michigan, USA; 3 Department of Atmospheric Sciences, School of Engineering, University of Michigan, Ann Arbor, Michigan, USA; 4 Departments of Medicine and Epidemiology, Columbia University Medical Center, New York, New York, USA; 5 Department of Preventive Medicine, Feinberg School of Medicine, Northwestern University, Chicago, Illinois, USA; 6 Department of Public Health Sciences, School of Medicine, Wake Forest University, Winston-Salem, North Carolina, USA; 7 Department of Environmental and Occupational Health Sciences, School of Public Health, University of Washington, Seattle, Washington, USA

**Keywords:** air pollution, blood pressure, cardiovascular disease, epidemiology, particulate matter

## Abstract

**Background:**

Blood pressure (BP) may be implicated in associations observed between ambient particulate matter and cardiovascular morbidity and mortality. This study examined cross-sectional associations between short-term ambient fine particles (particulate matter ≤ 2.5 μm in aerodynamic diameter; PM_2.5_) and BP: systolic (SBP), diastolic (DBP), mean arterial (MAP), and pulse pressure (PP).

**Methods:**

The study sample included 5,112 persons 45–84 years of age, free of cardiovascular disease at the Multi-Ethnic Study of Atherosclerosis baseline examination (2000–2002). Data from U.S. Environmental Protection Agency monitors were used to estimate ambient PM_2.5_ exposures for the preceding 1, 2, 7, 30, and 60 days. Roadway data were used to estimate local exposures to traffic-related particles.

**Results:**

Results from linear regression found PP and SBP positively associated with PM_2.5_. For example, a 10-μg/m^3^ increase in PM_2.5_ 30-day mean was associated with 1.12 mmHg higher pulse pressure [95% confidence interval (CI), 0.28–1.97] and 0.99 mmHg higher systolic BP (95% CI, –0.15 to 2.13), adjusted for age, sex, race/ethnicity, income, education, body mass index, diabetes, cigarette smoking and environmental tobacco smoke, alcohol use, physical activity, medications, atmospheric pressure, and temperature. Results were much weaker and not statistically significant for MAP and DBP. Although traffic-related variables were not themselves associated with BP, the association between PM_2.5_ and BP was stronger in the presence of higher traffic exposure.

**Conclusions:**

Higher SBP and PP were associated with ambient levels of PM_2.5_ and the association was stronger in the presence of roadway traffic, suggesting that impairment of blood pressure regulation may play a role in response to air pollution.

Exposure to ambient particulate matter (PM) has been associated with increased hospital admissions for cardiovascular disease, and increased cardiovascular disease mortality (Dockery 2001; Miller et al. 2007). Studies have suggested that vascular, autonomic, endothelial functioning and/or inflammation are part of the mechanistic pathway (Brook et al. 2002; Choi et al. 2007; Diez Roux et al. 2006; Gold et al. 2000; O’Neill et al. 2005); yet much of the evidence has been weak or inconclusive/null, or has come from small studies and/or among persons with preexisting disease. Thus, much remains to be learned about the underlying mechanisms or pathways of particle-induced mortality and morbidity. High blood pressure (BP) is an established risk factor for cardiovascular disease and may be implicated in the association between ambient PM and cardiovascular morbidity and mortality.

PM ≤ 2.5 μm in aerodynamic diameter (fine particles; PM_2.5_) can be effectively inhaled and deposited in the airways and alveolar surfaces, and thus have the potential to elicit health impacts [U.S. Environmental Protection Agency (EPA) 2004]. Inhaled particles may down-regulate nitric oxide synthase and alter autonomic nervous system functioning (Gold et al. 2000; Haak et al. 1994)—thus potentially affecting cardiac output, arterial stiffening, vascular function and tone, and wave reflections. Only one study (Zanobetti et al. 2004, a repeated-measures study among cardiac patients) has found a positive association between fine particles and BP. Most studies examining fine particles and BP have found inverse (Brauer et al. 2001; Ebelt et al. 2005; Ibald-Mulli et al. 2004) or no (Jansen et al. 2005; Linn et al. 1999; Urch et al. 2005) association. Studies to date have examined PM_2.5_ effects among persons with preexisting disease (chronic obstructive pulmonary disease or cardiac disease) or a small number of healthy individuals participating in an exposure-chamber study. Studies in general population samples are lacking. Furthermore, prior studies have investigated relatively short lags, ranging from same day to the 5 prior days, with one repeated-measures study examining 12-day exposure (Jansen et al. 2005).

We examined the cross-sectional relationship between relatively short-term exposure to PM_2.5_ (prior 1 day to prior 2 months) and BP in a large population-based sample. We hypothesized that recent exposure to PM_2.5_ would be positively associated with BP. We also hypothesized that proxies for traffic-related exposure would be associated with BP and would modify the association between background PM_2.5_ and BP, because near roadways PM_2.5_ may be higher and/or more toxic (Sanderson et al. 2005).

## Materials and Methods

### Study sample

Study participants, 45–84 years of age, from the Multi-Ethnic Study of Atherosclerosis (MESA) cohort were free of clinically apparent cardiovascular disease (symptoms, or history of medical or surgical treatment). They were recruited from six U.S. communities (Baltimore City and Baltimore County, Maryland; Chicago, Illinois; Forsyth County, North Carolina; Los Angeles County, California; Northern Manhattan and the Bronx, New York; and St. Paul, Minnesota) with a variety of population-based approaches, including commercial lists of area residents and random-digit dialing, as previously reported (Bild et al. 2002). In the present study we used data from the baseline visit occurring between July 2000 and August 2002; most (52%) participants were enrolled in 2001. The study was approved by each study site’s institutional review board. All participants provided written informed consent.

### Air pollution and meteorology data

We extracted pollutant data from the U.S. EPA’s Aerometric Information Retrieval System (U.S. EPA 2003). PM_2.5_ concentrations were obtained from 24-hr integrated samplers, which collected data daily or every third day. For each person, we created a set of PM_2.5_ concentrations representing exposure before the clinical examination based on concentrations from the monitor nearest to the person’s residence with available data on a given day. Five exposure measures were constructed: prior day, average of the prior 2 days, prior 7 days, prior 30 days, and prior 60 days.

Gaseous pollutants (sulfur dioxide, nitrogen dioxide, carbon monoxide), which are related to PM_2.5_ and potentially related to BP (Bhatnagar 2006), were also included. Ozone was not included because there was incomplete information in the winter. National Weather Service (National Climatic Data Center 2004) meteorologic variables (daily average temperature and sea-level barometric pressure) were included because of their strong associations with PM_2.5_ and BP (Woodhouse et al. 1993). Methods for co-pollutant and meterologic measures have been reported elsewhere (Diez Roux et al. 2006). Cumulative exposures for weather and co-pollutants were computed in the same way as PM_2.5_.

Proxies for traffic-related exposure were straight-line distance to a highway; total road length around the residence; and NO_2_, because it is a large component of traffic emissions (Brauer et al. 2002). The roadway file, obtained from the Environmental Systems Research Institute Inc. (ESRI, Redlands, CA), was a modified version of the 1990 Census TIGER/Line file. The modifications improved positional accuracy of the line file, eliminated errant segments, and appropriately reclassified/ corrected features [Geographic Data Technology (GDT) and ESRI 2002]. Residences within 300 m of a major road were defined as being “close to a highway.” Major roads were selected because they were likely to have diesel (truck) traffic (Brunekreef et al. 1997) and were identified from census feature class codes for primary roads (A1 or A2). A distance of 300 m was chosen for a few reasons: to obtain a reasonable distribution of residences in the two exposure categories, because traffic-related pollutants typically drop off to background levels around 300 m (Sanderson et al. 2005), and because potential spatial inaccuracies (Wu et al. 2005) in the relative positioning of our roadway data and participant addresses precluded accurate measures of very short distances. Total road length (major plus connecting roads) was calculated for a 400-m (0.25-mile) area around the residence and used as both a continuous and a binary variable (total road length Symbol 3.5 km, top quartile).

### Clinical measurements

We obtained information on person-level covariates during the clinical examination: age, sex, race/ethnicity, income, education, body mass index (BMI), type 2 diabetes [defined by the American Diabetes Association 2003 criteria (Genuth et al. 2003)], cigarette smoking, environmental tobacco smoke (ETS; during past year ≤ 1 hr/week in “close quarters” with a person who smoked at home, at work, in a car, etc.), high alcohol use (average weekly drinks was ≤ 7, top 10th percentile), sodium intake, physical activity, and BP medications. These variables are potential confounders because they are associated with BP outcomes and may be associated with residential location and therefore with PM and traffic exposures. We calculated per capita income by dividing the interval midpoint of family income (total combined family income for the preceding 12 months from 13 income categories, in U.S. dollars) by the number of persons supported. Dietary sodium intake was included in exploratory models (results were very similar), but not included in final models because a large proportion of data was missing. Antihypertension medication use was defined as using any of the common classes of antihypertensive medications: thiazide diuretics, β-blockers, calcium channel blockers, angiotensin-converting enzyme inhibitors, angiotensin-2 receptor blockers, and other α-blockers or peripheral vasodilators. (Results were very similar when medications were separated into classes of medications.)

Resting seated BP was measured three times at 1-min intervals using an automated oscillometric sphygmomanometer (Dinamap PRO 100; Critikon, Tampa, FL). The average of the second and third BP measurements was used for these analyses. The mechanisms through which PM exposures may affect BP (and hence the aspect of BP likely to be most sensitive to exposures) are unknown. We therefore examined a variety of BP parameters including systolic BP (SBP), diastolic BP (DBP), pulse pressure (PP; systolic–diastolic), and mean arterial pressure {MAP; [(2 × diastolic) + systolic]/3}.

Of the 6,814 participants who completed the clinical examination, 6,181 participated in the air pollution study. Of these persons, there were exclusions due to address errors (*n* = 149) or missing information on BP (*n* = 3), air pollution exposure (*n* = 661), and other covariates (*n* = 256). Therefore, data on 5,112 participants were available for analysis. The demographic characteristics of this subset were similar to those excluded (*n* = 1,702), except that excluded participants were less likely to be Caucasian (37% vs. 44%), had lower per capita family income ($21,700 vs. $26,400), were more likely to be from the St. Paul study site (St. Paul had a small number of PM monitors and infrequent data collection), and less likely to be from the Chicago site (8% vs. 21%).

### Statistical analysis

We used ordinary least-squares regression to separately estimate associations between BP and a 10-μg/m^3^ increase in PM_2.5_, using various PM_2.5_ averaging periods before and after adjustment for confounders: age, sex, race/ethnicity, per capita family income, education, BMI, diabetes status, cigarette smoking status, exposure to ETS, high alcohol use, physical activity, BP medication use, meteorology variables, and co-pollutants. Adjustment for confounders was performed in stages to identify which confounders had a strong influence on results.

Our statistical analyses relied on variability in particle concentrations between days and between study sites. Thus, adjustment for study site may reduce our ability to detect an association between particles and blood pressure. Nevertheless, because particle composition may vary by study site and may be associated with BP through mechanisms not already controlled for (Diez Roux et al. 2002; Lakoski et al. 2005), we also examined associations after adjustment for site as well as heterogeneity in associations by site. We examined associations between BP and traffic-related variables using the same sequential modeling approach used for PM_2.5_.

We investigated heterogeneity in the association between PM_2.5_ and BP by levels of traffic related exposures [living close (≤ 300 m) to a highway, surrounded by a high density of roads, high NO_2_ exposure], for levels of SO_2_ and CO, and for weather variables. “High” levels were defined as the top quartiles of these variables. Heterogeneity of effects was also examined by age, sex, type 2 diabetes, hypertensive status, and cigarette use. Older persons, women (Künzli et al. 2005; Miller et al. 2007), and people with diabetes (O’Neill et al. 2005) and hypertension may be more vulnerable to the effects of air pollution; and direct inhalation of PM from cigarettes may overwhelm any effects because of ambient particle exposure. We tested heterogeneity of effects by stratification and by including interaction terms in regression models.

Because BP medications potentially have a strong influence on continuous BP, medication use was controlled for; heterogeneity of effects by medication use was examined; and secondary analyses used log binomial models to fit a binary hypertension outcome (any of the following: SBP ≥ 140 mmHg, DBP ≥ 90 mmHg, self reported history of hypertension, use of hypertensive medication) (Chobanian et al. 2003).

We assessed nonlinear covariate-adjusted relationships between all independent variables and BP outcomes (Hastie 1992). There was no evidence of strong threshold/nonlinear effects for PM_2.5_ although nonlinearity was evident among co-pollutants; thus, we fit ordinary least squares regression with piecewise linear functions as appropriate.

We confirmed that multivariable regression variance inflation factors (VIF) did not diagnose high collinearity; VIF = 10 was used to define high multicollinearity (Belsley et al. 1980; Kleinbaum et al. 1998).

Because seasons vary by our study sites, primary analyses used temperature and barometric pressure to adjust for seasonality. Sensitivity analyses evaluated whether there was seasonal residual confounding or autocorrelation not accounted for by weather variables. Main results were stratified by season. Results were also examined before and after adding a smoothing spline [in generalized additive models (Hastie 1992)] for season. We assessed autocorrelation among regression residuals via autoregressive generalized estimation models (Littell et al. 1996), by examining the Durbin–Watson statistic in ordinary least-squares regression (Durbin and Watson 1951), and plotting residuals against time (time represented BP collection year–month and also season).

Sensitivity analyses also evaluated the robustness of results to exposure misspecification. Analyses using PM_2.5_ were repeated *a*) restricting analyses to participants living relatively close (20 km) to PM_2.5_ monitors; and *b*) stratifying results by the years the participant lived at the address and the amount of time each week spent within that neighborhood. We also repeated analyses for proximity of highways using continuous distance to highways and an alternate binary measure (≤ 400 m from a highway).

## Results

Mean age in the study sample was 62 years, approximately half were female, mean BMI was 28 kg/m^2^, 45% had hypertension, and 38% were taking BP medications (Table 1). Prior 30-day mean PM_2.5_ was highly correlated with prior 60-day mean PM_2.5_ (Spearman *r* = 0.87) but more weakly correlated with other time periods [*r* = 0.67, 0.48, and 0.41, for prior 7-, 2-, and 1-day(s), respectively] [see Supplemental Material (online at http://www.ehponline.org/members/2008/10899/suppl.pdf) for more correlations]. Mean 30-day PM_2.5_ was highest in Los Angeles (21.8 μg/m3) and lowest in St. Paul (10.3 μg/m^3^), and 29% of the sample lived close to a highway. Mean PM_2.5_ was much higher where NO_2_ was high (19.9 vs. 15.7 μg/m^3^) but PM_2.5_ levels varied little with other traffic-related variables. Patterns were similar for prior 1-, 2-, 7-, and 60-day exposures (data not shown.)

Table 2 shows adjusted associations of PM_2.5_ with BP outcomes. We present results only for PP and SBP. None of the DBP results were statistically significant. Results for MAP were similar to SBP, though weaker and generally not statistically significant. Results for DBP and MAP are shown in Supplemental Material (online at http://www.ehponline.org/members/2008/10899/suppl.pdf) (results for DBP can be derived by subtracting the PP from SBP values shown in Table 2). PP and SBP were generally associated with individual-level covariates in the expected direction, and meteorology variables (temperature and atmospheric pressure) were positively associated with BP (data not shown). Adjusted for age, sex, race/ethnicity, income, education, BMI, diabetes, cigarette smoking, alcohol use, physical activity, BP medication, and meteorology variables, a 10-μg/m^3^ increase in PM_2.5_ was associated with 1.12 mmHg higher PP [95% confidence interval (CI), 0.28–1.97] and 0.99 mmHg higher SBP [although CIs included the null value (95% CI, –0.15 to 2.13), model 2]. Results were noticeably stronger after adjustment for gaseous co-pollutants [per 10-μg/m^3^ increase in PM_2.5_, PP was 2.66 higher (95% CI, 1.61–3.71] and SBP was 2.80 higher (95% CI, 1.38–4.22), model 2a]; the impact was greatest after adding NO_2_ to the models [Supplemental Material (online at http://www.ehponline.org/members/2008/10899/suppl.pdf) shows regression estimates for the co-pollutants]. Adding site to model 2 had no effect on PP, but strengthened the SBP results while widening CIs [per 10-μg/m^3^ increase in PM_2.5_ there was a 1.32 (95% CI: –0.18 to 2.82) increase in SBP, model 3a]

Associations between PM _2.5_ and BP became stronger with longer PM_2.5_ averaging periods up to 30 days. For example, per 10-μg/m^3^ difference in PM_2.5_ adjusted for covariates in model 2, the difference in PP was –0.38 mmHg for 1 day (95% CI, –0.76 to 0.00); –0.22 mmHg for 2 days (95% CI, –0.65 to 0.21); 0.52 mmHg for 7 days (95% CI, –0.08 to 1.11); 1.12 mmHg for 30 days (0.28 to 1.97); and 1.08 mmHg for 60 days (95% CI, 0.11 to 2.05). This pattern held true for other person-level adjustments and for SBP, so only results for the 30-day mean differences are shown [see Supplemental Material (online at http://www.ehponline.org/members/2008/10899/suppl.pdf) for the 60-day averages].

We examined comparable models for traffic-related exposures. Associations with BP were opposite expectation (negative) and generally statistically significant [see Supplemental Material (online at http://www.ehponline.org/members/2008/10899/suppl.pdf) for results]. After adjustment for study site, the magnitude of the associations decreased, and most CIs included the null value. Results were similar when adjusted for ambient PM_2.5_ exposure.

**Figure 1:**
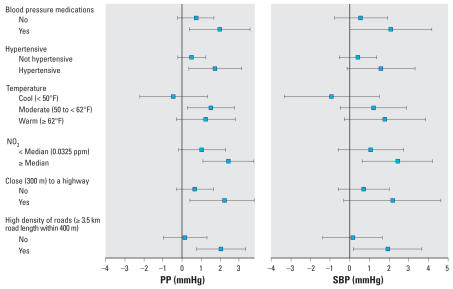
Stratified estimates (95% CIs) of the difference in PP and SBP per 10-μg/m^3^ increase in PM_2.5_ prior 30-day average, adjusted for model 2 covariates (except the stratification by temperature, which was adjusted for model 1 covariates). All tests for interactions were *p* < 0.09 except for Close to a highway: *p* < 0.3 for SBP.

The associations of PM_2.5_ with BP were not modified by age, sex, diabetes, cigarette use, study site, high levels of CO or SO_2_, season, nor residence ≤ 400 m from a highway (tests for interaction, all *p ≥* 0.2). Figure 1 shows variables for which interactions were *p* ≤ 0.1 for either PP or SBP [see Supplemental Material (online at http://www.ehponline.org/members/2008/10899/suppl.pdf) for all heterogeneity analyses]. Associations between PM_2.5_ and BP were stronger for persons taking medications, with hypertension, during warmer weather, in the presence of high NO_2_, residing ≤ 300 m from a highway, and surrounded by a high density of roads.

Sensitivity analyses that examined results restricted to persons who lived relatively close (≤ 20 km) to their PM_2.5_ monitors found similar (though stronger) results, and inference generally remained the same. Results were insensitive to the number of years spent living in the residence and the percentage of time during a week spent in the neighborhood. (On average, participants lived 15 years in their neighborhood and spent 75% of their time within their neighborhood.)

There was no strong evidence of seasonal residual autocorrelation: the Durbin–Watson statistic was normal, and plots of residuals against time (time represented season and BP collection year–month) did not show strong patterning. Results were very similar when a smoothing spline for season was added to the model and when structured covariance models allowed for autocorrelation between months [see Supplemental Material (online at http://www.ehponline.org/members/2008/10899/suppl.pdf)].

Secondary analyses that replaced the continuous BP outcome with binary hypertension (using log binomial models) found results that were mostly positive though somewhat weaker and often included the null value (data not shown).

## Discussion

In this cross-sectional study, PP and SBP were positively associated with recent ambient levels of PM_2.5_ at the participant’s residence. Associations between PM_2.5_ and PP persisted after adjustment for individual level confounders, as well as other environmental variables. PP was 1.12 mmHg higher (95% CI, 0.28–1.97) for each 10-μg/m^3^ increase in prior 30-day mean PM_2.5_ adjusted for person-level confounders, atmospheric pressure, and temperature. A 10-μg/m^3^ increase was approximately equivalent to the difference in prior 30-day mean PM_2.5_ between the 90^th^ and 10th percentile. Associations between PM_2.5_ and SBP were generally weaker except when gaseous co-pollutants were also adjusted for: SBP was 2.80 mmHg higher (95% CI, 1.38–4.22) per 10-μg/m^3^ increase in prior 30-day mean PM_2.5_. In addition, associations between particles and BP were stronger in the presence of traffic-related measures.

Our results (for SBP, model 2a) were roughly comparable to the only other study that found a positive association between PM_2.5_ and BP: Zanobetti et al.’s (2004) repeated-measures study among cardiac patients which derived exposure from prior 5-day mean PM_2.5_, and controlled for age, sex, BMI, number of visits, hour of the day, and weather. An advantage of our study over prior work is improved generalizability. Our sample was large and geographically and demographically diverse, and participants were generally healthy. All (non-chamber) studies examining fine particle effects on BP were among persons with preexisting disease, and nearly all studies used a single geographic area (Brauer et al. 2001; Ebelt et al. 2005; Jansen et al. 2005; Linn et al. 1999; Zanobetti et al. 2004) except Ibald-Mulli et al.’s study of three cities in northern Europe (Ibald-Mulli et al. 2004). Most previous work examining the relationship between BP and PM_2.5_ has investigated relatively acute exposures. We found stronger effects from longer (30- and 60-day) averages of ambient PM_2.5_ exposure compared with shorter (1-, 2-, 7-day) averages, as has been found for other health outcomes (Diez Roux et al. 2006). Accumulated exposure may have a greater impact on health, or smoothing across wide fluctuations in PM levels may reduce noise and hence yields better estimates of (even recent) true background exposure.

The magnitude of the PM_2.5_–BP associations we found were roughly equivalent to a cross-sectional increase in PP and SBP associated with aging 1.5 to 3.5 years in our sample. Although associations of this magnitude are relatively small, they nevertheless provide some evidence that BP (or pathways controlling vascular homeostasis) might play a mediating role in the association already observed between particles and cardiovascular morbidity and mortality. There has been growing interest in the role of PP as a risk factor for cardiovascular disease (especially among older adults) (Assmann et al. 2005; Benjo et al. 2007), and we found that among BP-related outcomes, associations between PM_2.5_ and PP tended to be strongest and/or statistically significant. This may suggest that, in our sample of older adults, PM_2.5_ relates more to pulsatile stress of large-artery stiffness and impairment of vascular tone than to other mechanisms in BP disorders (O’Rourke and Mancia 1999). Although still positive, in some models there were weaker results for SBP which may suggest that cardiac ejection is a less important mechanism than vascular distensibility and tone. Particles’ potential impairment of vascular tone is supported by prior work suggesting that particle inhalation may trigger endothelial dysfunction (Brook et al. 2002; O’Neill et al. 2005; Tornqvist et al. 2007).

A key mechanism by which particles could trigger vascular dysfunction is by down-regulating NO synthase. For example, inhalation of particles via cigarette smoking inhibits endogenous NO production (Bhatnagar 2006; Malinovschi et al. 2006). Although reduced bioavailability of NO contributes to alterations in BP-related functions (e.g., endothelial function, activation of the sympathetic system, platelet functioning) (Bhatnagar 2006; Haak et al. 1994; Rajagopalan et al. 2005), reductions in NO also increase the instability of BP which is itself a risk factor for cardiovascular disease (Mancia et al. 2001; Stewart et al. 1994). Thus, measures of short-term intraindividual BP variability could reveal effects that our measures at a single point in time do not. Future work could extend this study to a repeated-measures design permitting improved control for person-level factors and examination of within-person variability of BP.

Like previous population-based studies that examined associations between BP and air pollution (Harrabi et al. 2006; Ibald-Mulli et al. 2001), our study included persons taking BP medications. Medication use is presumably both a predictor of lower BP and predicted by BP (medication is prescribed when pressure is elevated), and thus potentially participates in a recursive feedback mechanism that is impossible to model accurately using traditional statistical methods (Wang 2006). Our results were robust to adjustment for medication use and other related variables (age, race/ethnicity, income, and education) and when hypertension was used as an outcome variable. The stronger association between particle exposure and BP among hypertensives and medication users may suggest that these persons are more vulnerable to air pollution effects, as found in previous studies (Frank and Tankersley 2002; O’Neill et al. 2005).

We found no evidence of a main local traffic association with BP, although local traffic exposures modified the association between background PM_2.5_ and BP. The lack of an association with BP may have been attributable to traffic exposure measurement error. We were not able to assess closer roadway exposure [few participants resided within 50 or 100 m of highways, and there were potential spatial inaccuracies in the relative positioning of our roadway data and participant addresses (Wu et al. 2005)], nor differentiate traffic sources most toxic [e.g., diesel (Brunekreef et al. 1997)]. The stronger association between background PM_2.5_ and BP in the presence of greater exposure to traffic may have been attributable to compositional differences in PM_2.5_ near roadways—including a more toxic mixture of pollutants. A greater proportion of particles near roadways are ultrafine (< 0.1 μm) (Sanderson et al. 2005), which are most detrimental to health (Nemmar et al. 2002; U.S. EPA 2004). In addition, non-PM_2.5_ pollutants from roadways may potentiate the PM_2.5_–BP effect: We found stronger associations in the presence of high NO_2_. Differences in particle composition may have also contributed to stronger (positive) associations between PM_2.5_ and BP during moderate/warm weather, as was seen in a previous study of PM_10_ (PM with aero-dynamic diameter ≤ 10 μm) and BP (Choi et al. 2007). Photochemical conditions can increase certain copollutants (e.g., ozone is highest during warm weather) and pollution sources may seasonally vary (e.g., vehicular traffic or types of fuel being burned) (U.S. EPA 2004). In addition, in moderate/warm weather, participants may increase their exposure to ambient PM through time spent outdoors and indoor ventilation from open windows (Zeka et al. 2006).

Associations between PM_2.5_ and BP were positive and then became much stronger after adjustment for SO_2_, NO_2_, and CO. Although studies have generally found an unchanged or weakened effect of PM_2.5_ on health after adjustment for copollutants (e.g., Zanobetti et al. 2004), a slightly strengthened effect has been found in some studies (e.g., Miller et al. 2007). Our results were likely strengthened because of strong negative confounding by NO_2_ and (to some extent) by SO_2_. An alternate explanation is that the PM_2.5_ effect after co-pollutant adjustment was upwardly biased because of strong negative correlations among co-pollutant measurement errors (Zeger et al. 2000).

We identified PM_2.5_ exposure using the monitor nearest each participant’s residence. Our method is likely an improvement over using a central-city or health clinic monitoring site, which are often used in air pollution studies (Ibald-Mulli et al. 2004; Zanobetti et al. 2004). Results were generally insensitive to participant distance to monitors, likely because there was high within-site correlation of background PM_2.5_ and because most participants spent much of their time near their home. Nevertheless, we acknowledge that our exposures did not account for within-day exposure variability (e.g., while commuting, at the workplace) nor indoor particulate levels in general. In defense of the measures we used, in the United States ambient PM_2.5_ originating from outside sources appears to be more toxic to health than PM_2.5_ originating from indoor sources (Ebelt et al. 2005). Moreover, our results were adjusted for smoking and ETS, which are likely major contributors to indoor exposures in the population we studied.

In summary, recent exposure to PM_2.5_ was positively associated with PP, and the association was stronger for residents with higher exposure to traffic-related measures. Our results suggest that BP—particularly arterial stiffness and reductions in vascular tone—may play a mediating role in associations already observed between particles and cardiovascular morbidity and mortality. Given the distal pathway between environmental exposures and their potential pathologic effects, it is noteworthy that we found even a modest association. Because ambient air pollution is ubiquitous, small effects have the potential to substantially affect public health. This study provides supportive evidence that reducing population-level exposures to ambient pollution can potentially improve population health.

## Figures and Tables

**Table 1:** Demographic characteristics, BP, and environmental characteristics, MESA 2000–2002 (*n* = 5,112).

Variable	Value	PM_2.5_ prior 30 days (mean ± SD)
Demographic characteristics		
Age [years (mean ± SD)]	62.3 ± 10.0	
Female (%)	52	
Race/ethnicity (%)		
Caucasian	37	
Chinese	14	
African American	28	
Hispanic	21	
Per capita income [per $1,000 (mean ± SD)]	27 ± 21	
No college education (%)	34	
BMI [kg/m^2^ (mean ± SD)]	28.2 ± 5.4	
Diabetes (%)		
No diabetes or impairment	58	
Impaired glucose tolerance	29	
Diabetes	14	
Smoking status (%)		
Never	51	
Former	37	
Current	12	
Environmental tobacco smoke (%)		
≥ 1 hour per week	33	
High alcohol use (%)		
High (top 10th percentile, ≥ 7 drinks per week)	12	
Physical activity^a^		
Low	28	
Medium	49	
High	23	
Taking medications for blood pressure (%)	38	
Blood pressure		
Systolic blood pressure [mmHg (mean ± SD)]	126.5 ± 21.0	
Dystolic blood pressure [mmHg (mean ± SD)]	72.0 ± 10.2	
Pulse pressure [mmHg (mean ± SD)]	54.4 ± 16.9	
Arterial pressure (mean ± SD)	90.2 ± 12.4	
Hypertension^b^ (%)	45	
Environmental characteristics		
PM_2.5_ [μg/m^3^ (mean ± SD)]^c^
Prior day	17.0 ± 10.5	
Prior 2 days	16.8 ± 9.3	
Prior 7 days	17.0 ± 6.9	
Prior 30 days	16.8 ± 5.0	
Prior 60 days	16.7 ± 4.4	
Study site (%)		
Los Angeles County, CA	22	21.8 ± 5.4
Chicago, IL	20	16.7 ± 3.9
Baltimore, MD	17	15.9 ± 3.6
St. Paul, MN	8	10.3 ± 2.4
Forsyth County, NC	16	15.4 ± 3.3
Northern Manhattan and Bronx, NY	18	15.4 ± 2.9
Traffic-related^d^		
Close to a highway (%)		
No	71	16.9 ± 5.2
Yes	29	16.5 ± 4.3
Surrounded by a high density of roads (%)		
No	75	16.9 ± 5.2
Yes	25	16.5 ± 4.1
NO_2_, prior 30 days (%)		
Low	75	15.7 ± 4.1
High	25	19.9 ± 5.9

a Reported total physical activity classified based on the lowest and highest quartiles: low < 9 hr/day, medium 9–16 hr/day, high > 16 hr/day.

b Having any of the following: DBP ≥ 90, SBP ≥ 140, self-reported history of hypertension, use of hypertensive medication (Chobanian et al. 2003).

c The 2006 National Ambient Air Quality Standards for PM_2.5_: 15 μg/m^3^ for annual mean and 35 μg/m^3^ for 24-hr mean (U.S. EPA 2006)

d Close to a highway was ≤ 300m of a major road. Surrounded by a high density of roads was defined as ≥ 3.5 km (top quartile) of road length within 400 m of the residence. High NO_2_ was defined as 0.0325 ppm (top quartile). This cut point for NO_2_ is much lower than the annual NAAQS of 0.053 ppm.

**Table 2:** Adjusted mean differences (95% CIs) in PP and SBP (mmHg) per 10-ug/m^3^ increase in PM_2.5_ (averaged for the prior 1–30 days) (*n* = 5,112), MESA, 2000–2002.

		PP			SBP		
Model no.	Adjustment variables	Mean difference	95% CI	p-Value	Mean difference	95% CI	p-Value
1	Person-level covariates^a^	1.04	0.25 to 1.84	0.010	0.66	−0.41 to 1.74	0.226
2	Person-level covariates,^a^ weather^b^	1.12	0.28 to 1.97	0.009	0.99	−0.15 to 2.13	0.089
2a	Person-level covariates,^a^ weather,^b^ gaseous co-pollutants^c^	2.66	1.61 to 3.71	0.000	2.8	1.38 to 4.22	0.000
3	Person-level covariates,^a^ study site	0.93	−0.04 to 1.90	0.060	0.86	−0.45 to 2.17	0.200
3a	Person-level covariates,^a^ study site, weather^b^	1.11	0.01 to 2.22	0.049	1.32	−0.18 to 2.82	0.085
3b	Person-level covariates,^a^ study site, weather,^b^ gaseous co-pollutants^c^	1.34	0.10 to 2.59	0.035	1.52	−0.16 to 3.21	0.077

Adjusted relationships between blood pressure and temperature and SO_2_ were fit using piecewise linear splines because they are positive for lower values and negative for higher values (breaks at 45ºF for temperature and 0.004 ppm for SO_2_).

a Age, sex, race/ethnicity, per capita income, education, BMI, diabetes status, cigarette smoking, exposure to ETS, alcohol use, physical activity, medications.

b Prior 30-day mean for temperature and sea-level pressure.

c Prior 30-day mean for NO_2_, SO_2_, and CO.
